# Longitudinal survey of *Clostridium difficile* presence and gut microbiota composition in a Belgian nursing home

**DOI:** 10.1186/s12866-016-0848-7

**Published:** 2016-10-01

**Authors:** Cristina Rodriguez, Bernard Taminiau, Nicolas Korsak, Véronique Avesani, Johan Van Broeck, Philippe Brach, Michel Delmée, Georges Daube

**Affiliations:** 1 Food Science Department, FARAH, Faculty of Veterinary Medicine, University of Liège, Liège, Belgium; 2 National Reference Laboratory for Clostridium difficile, Cliniques Universitaires Saint Luc, Microbiology Unit, Catholic University of Louvain, Brussels, Belgium; 3 Nursing Home Saint-Joséphine site de la Chaussée, ACIS, Theux, Belgium

**Keywords:** *C. difficile*, Elderly care home residents, 16S rRNA gene analysis

## Abstract

**Background:**

Increasing age, several co-morbidities, environmental contamination, antibiotic exposure and other intestinal perturbations appear to be the greatest risk factors for *C. difficile* infection (CDI). Therefore, elderly care home residents are considered particularly vulnerable to the infection. The main objective of this study was to evaluate and follow the prevalence of *C. difficile* in 23 elderly care home residents weekly during a 4-month period. A *C. difficile* microbiological detection scheme was performed along with an overall microbial biodiversity study of the faeces content by 16S rRNA gene analysis.

**Results:**

Seven out of 23 (30.4 %) residents were (at least one week) positive for *C. difficile. C. difficile* was detected in 14 out of 30 diarrhoeal samples (43.7 %). The most common PCR-ribotype identified was 027. MLVA showed that there was a clonal dissemination of *C. difficile* strains within the nursing home residents. 16S-profiling analyses revealed that each resident has his own bacterial imprint, which was stable during the entire study. Significant changes were observed in *C. difficile* positive individuals in the relative abundance of a few bacterial populations, including *Lachnospiraceae* and *Verrucomicrobiaceae.* A decrease of *Akkermansia* in positive subjects to the bacterium was repeatedly found.

**Conclusions:**

A high *C. difficile* colonisation in nursing home residents was found, with a predominance of the hypervirulent PCR-ribotype 027. Positive *C. difficile* status is not associated with microbiota richness or biodiversity reduction in this study. The link between *Akkermansia*, gut inflammation and *C. difficile* colonisation merits further investigations.

**Electronic supplementary material:**

The online version of this article (doi:10.1186/s12866-016-0848-7) contains supplementary material, which is available to authorized users.

## Background

*Clostridium difficile* is a Gram-positive, anaerobic, spore-forming, rod-shaped bacterium that has been widely described in the intestinal tract of humans and animals. In 1978, *C. difficile* was recognized as a major cause of antibiotic associated diarrhoea and, in the most serious cases pseudomembranous colitis [1–3]. Since then, many outbreaks have been reported; most of them were associated with the emergence of a specific subtype, hyper-virulent PCR-ribotype 027 [4]. Nowadays, *C. difficile* is a worldwide public health concern as it is considered the major cause of antibiotic-associated infections in healthcare settings [5]. A recent report of *C. difficile* infection (CDI) cost-of-illness attributes a mean cost ranging from 8,911 to 30,049 USD for hospitalised patients (per patient/admission/episode/infection) in the USA [6] and annual economic burden estimated around 3,000 million euro in Europe [7].

CDI is more commonly diagnosed among older people in nursing homes. High isolation frequencies have been described in USA, with up to 46 % of elderly residents testing positive for *C. difficile*, while in Europe or Canada the reported rates are much lower, varying between 0.8 and 10 % [8]. This is partly because elderly people are more commonly in hospitals, have an antibiotic treatment and age-related changes in intestinal flora and host defences, as well as the presence or other underlying health problem [8–10]. These factors can have an impact on the intestinal microbiota, which may promote *C. difficile* colonisation and the development of the infection [11]. Therefore, a new concern of several studies has been the identification of the microbial communities implicated in the CDI through the use of new sequencing techniques, like metagenomics [12].

The aim of this study was to evaluate and follow the prevalence of *C. difficile* among the residents of a Belgian nursing home. Multilocus variable number of tandem repeats analysis (MLVA) was performed to determine the genetic diversity of the *C. difficile* isolates and possible cross-infection between patients. Additionally, 16S rRNA gene sequencing was used to characterise the faecal microbiota of the elderly residents, to evaluate the global evolutions of the total microbiota and to identify possible relationships between certain bacteria populations and *C. difficile* colonisation, diarrhoea and antibiotic treatment.

## Results

### Prevalence of *C. difficile*

A total of 242 faecal samples were collected from 23 residents in seventeen consecutive weeks (resident number 11 was excluded from the study as he finally did not agree to participate in the survey). Two subjects passed away within the four-month study period. Seven out of 23 monitored residents were positive for *C. difficile* at least once (Table 1).

**Table 1:** Detailed information on 23 nursing home residents enrolled in the study, including the detection of *C. difficile* with and without enrichment

Resident identification	Week																
	1	2	3	4	5	6	7	8	9	10	11	12	13	14	15	16	17
01	E	E	‡	-	-	‡	-	-	‡	E	E	E	‡	D	‡	D	-
02	-	-	-	-	-	-	-	-	-	-	-	-	-	-	-	-	-
03	-	-	-	‡	-	-	-	-	-	‡	-	-	‡	-	‡	‡	‡
04	-	-	-	-	-	-	-	-	-	-	-	‡	-	-	-	-	-
05	-	-	-	‡	‡	-	‡	‡	-	-	-	-	‡	-	-	-	‡
06	-	-	-	‡	-	‡	-	-	-	-	‡	-	‡	‡	-	-	‡
07	-	-	-	‡	‡	-	-	-	-	‡	-	-	-	-	‡	-	‡
08	‡	-	-	H	H	H	H	-	‡	-	-	-	-	‡	-	‡	‡
09	-	‡	‡	-	‡	‡	‡	-	-	-	‡	-	-	-	‡	-	-
10	-	‡	-	‡	-	-	‡	-	-	-	-	-	‡	-	-	‡	‡
12	-	-	-	-	‡	-	-	-	‡	-	-	-	-	-	‡	-	-
13	D	D	-	-	‡	-	-	-	‡	-	-	-	‡	-	‡	‡	‡
14	-	-	-	-	‡	‡	‡	-	-	-	‡	-	‡	-	‡	‡	‡
15	E	E	E	-	‡	D	D	D	D	D	D	D	D	D	‡	‡	D
16	-	-	-	-	-	-	†	†	†	†	†	†	†	†	†	†	†
17	-	-	-	‡	-	-	-	D	-	-	-	-	‡	-	‡	‡	‡
18	‡	‡	‡	‡	D	‡	D	‡	D	‡	‡	D	‡	‡	E	‡	E
19	E	E	E	‡	‡	‡	D	‡	-	-	E	‡	D	‡	-	‡	‡
20	-	‡	-	‡	‡	-	‡	‡	-	‡	‡	‡	‡	‡	-	‡	‡
21	‡	-	-	-	-	-	‡	-	‡	-	-	‡	‡	‡	-	‡	-
22	-	-	-	-	-	-	†	†	†	†	†	†	†	†	†	†	†
23	-	‡	-	-	-	‡	-	-	-	-	-	-	-	‡	-	-	-
24	‡	‡	D	‡	E	-	‡	E	‡	-	‡	-	‡	-	‡	‡	-

Resident number 11 was excluded from the study

D: Positive results detected without enrichment

E: Positive results detected after 3 days of enrichment

-: Negative results for *C. difficile* presence

**‡**: Sample was not available

H: resident hospitalized

**†**: The resident passed away during the study period

There was only one case of CDI diagnosed during the study (subject 01). He was diagnosed in week eleven of the study after suffer more than three episodes consecutives of diarrhoea. *C. difficile* was detected in 14 out of 30 diarrhoeal samples (43.7 %). Regarding the antimicrobial therapy, a total of five residents tested positive for *C. difficile* had previously received an antibiotic medication. Probiotic treatment was noted in 4 residents, two of them were positive for *C. difficile*. Only one resident (number 08) was hospitalized during the study (Table 2).

**Table 2:** Clinical characteristics of the 23 residents enrolled in the study and molecular type of the isolates

Resident number	Age (years)	Genre	Status	Room floor	Diarrhea	Hospital stay	Antibiotic treatment	Probiotic treatment	*C. difficile* culture	PCR-ribotype	N° isolates	CE	*tcdA tcdB*	*cdtA cdtB*	*tcdC* MUT	*gyrA* MUT
01	77	M	SD	1	+	-	+	-	+	UCL16a	9	+	+	-	-	-
02	78	F	D	2	-	-	-	-	-	-	-	-	-	-	-	-
03	92	F	D	2	-	-	-	-	-	-	-	-	-	-	-	-
04	88	F	D	1	+	-	-	-	-	-	-	-	-	-	-	-
05	93	F	D	1	+	-	+	+	-	-	-	-	-	-	-	-
06	86	F	D	2	-	-	+	-	-	-	-	-	-	-	-	-
07	92	F	SD	3	-	-	+	-	-	-	-	-	-	-	-	-
08	91	F	SD	3	-	+	+	+	-	-	-	-	-	-	-	-
09	88	F	D	2	-	-	-	-	-	-	-	-	-	-	-	-
10	78	F	SD	2	-	-	-	-	-	-	-	-	-	-	-	-
12	87	F	D	2	+	-	-	-	-	-	-	-	-	-	-	-
13	65	M	D	1	-	-	+	-	+	UCL36	4	-	-	-	-	-
14	76	F	SD	1	-	-	-	-	-	-	-	-	-	-	-	-
15	50	F	D	2	+	-	-	+	+	027	22	+	+	+	+	+
16	94	F	D	3	+	-	+	-	-	-	-	-	-	-	-	-
17	63	F	D	3	+	-	+	-	+	UCL36	1	-	-	-	-	-
									+	027	1	+	+	+	+	+
18	86	M	D	2	+	-	-	-	+	027	10	+	+	+	+	+
19	89	F	SD	3	+	-	+	-	+	UCL36	2	-	-	-	-	-
										UCL46	2	+	+	-	-	-
										027	4	+	+	+	+	+
20	81	F	SD	1	-	-	+	-	-	-		-	-	-	-	-
21	82	F	D	1	+	-	-	-	-	-		-	-	-	-	-
22	83	F	D	1	-	-	-	-	-	-		-	-	-	-	-
23	88	F	D	2	-	-	-	-	-	-		-	-	-	-	-
24	81	F	D	1	-	-	+	+	+	UCL36	4	-	-	-	-	-

*M* male

*F* female

*SD* semi-dependant residents

*D* dependant residents

*CE* cytotoxicity assay using MRC-5 cells

*tcdC* MUT: Presence of deletions in the regulator gene *tcdC* (118 bp-39 bp-17 bp)

*gyrA* MUT: Presence of mutation in the *gyrA* gene associated with moxifloxacin resistance

### Characterization of *C. difficile* isolates

Four different PCR-ribotypes (UCL16a, UCL36, UCL46 and 027) were identified among the 38 isolates. In one resident (number 19), different PCR-ribotypes were found in different sampling days while in another subject (number 17) two different PCR-ribotypes were detected in the same sampling day (direct culture: PCR-ribotype 027; 3 days of stool enrichment: PCR-ribotype UCL36). Only in one resident (number 015), all but one samples obtained were positive for *C. difficile* and the isolated strains were all identified as PCR-ribotype 027. Three out of these four different PCR-ribotypes had toxin activity. All toxigenic isolates encoded toxin A and B, while PCR-ribotype 027 also contained the binary toxin. In addition, all types 027 contained an 18-base pair deletion, a deletion at 117 of the *tcdC* gen and *gyrA* mutation associated with moxifloxacin resistance (Table 2).

### *C. difficile* MLVA analysis

MLVA was performed in order to provide further insight into the clonal relatedness of the *C. difficile* isolates and cross-infection between patients. A total 59 isolates were obtained during the study. Among them, 44 toxigenic and non-toxigenic isolates were further analysed by MLVA. Selection of these strains was based on the inclusion of a representative number of isolates from each classified PCR ribotype. In order to determine if the seven variable-number-tandem repeat (VNTR) loci were stable over time or if subjects harboured more than one *C. difficile* type, isolates obtained from the same resident on direct culture and after 3 enrichment days and on different weeks were also studied by MLVA. Thirty-one different MLVA profiles were identified. However, a high degree of genetic relatedness was observed among most of the strains with the same PCR-ribotype (summed tandem repeat difference at all loci ≤ 2). The C6 and A6 were the most diverse VNTR loci. Regarding the strains identified as PCR-ribotype 027, most of them were closely related. Furthermore, several isolates from patients 15, 18 and 19 had an identical MLVA profile (Table 3).

**Table 3:** MLVA profile of the isolates obtained from each nursing home resident

PCR-Ribotype	MLVA profile								Resident number	No. of isolates	Week
	A6	B7	C6	E7	G8	CDR5	CDR60	Total			
027	22	9	38	10	17.5	3.9	7.2	107.6	15	4	1^E^ 2^E^ 6^E^ 7^E^
									18	1	
											5^E^
	23	9	38	10	17.5	3.9	7.2	108.6	15	1	3^E^
	22	9	39	10	17.5	3.9	7.2	108.6	15	1	7^D^
	22.2	9	37.8	10	17.5	3.9	7.2	107.6	15	3	9^D^ 10^E^ 14^E^
									18	1	
											15^E^
									19	1	12^D^
	22.2	9	37.8	10	17.5	3.8	7.2	107.5	15	1	8^E^
	22	9	37.8	10	17.5	3.8	7.2	107.3	15	1	9^E^
	23	9	37.8	10	17.5	3.8	7.2	108.3	15	1	12^D^
	22.2	9	26.5	10	18.5	3.9	7.2	97.3	15	1	12^E^
	23	9	40	10	17.5	3.9	7.1	110.5	18	1	2^E^
	22	9	36.8	10	17.5	3.9	7.2	106.4	18	1	7^D^
	22.2	9	37.8	10	17.6	3.9	7.2	107.7	18	1	7 ^E^
	23	9	36.8	10	17.5	3.8	7.2	107.3	18	1	9 ^E^
	22	9	37	10	17.5	3.9	7.2	106.6	19	1	3 ^E^
	22.2	9	36.8	10	17.5	3.8	7.2	106.5	19	2	7 ^E^ 11^E^
UCL16a	30.8	14.1	23.5^a^	5	10.8	6.8	3.2	94.2	1	1	1^E^
	30.8	14	23.5	5	10.8	6.8	3.2	94.1	1	2	2^E^ 16^E^
	30.8	14	24.5	5	10.8	6.8	3.2	95.1	1	1	10^E^
	30.7^b^	14^c^	11.3	5	10.8	6.8	3.2	81.8	1	1	11^E^
	29.8	14	23.5	5	10.8	6.8	3.2	93.1	1	2	12^E^ 16^D^
	31.8^d^	14	23.5	5	10.8	6.8	3.2	95.1	1	1	14^D^
UCL46	28.8	21.1	22.3	14	8	8.8	2.2	105.2	19	1	1^E^
	28.8	21.1	22.5	14	8	8.8	2.3	105.5	19	1	2^E^
UCL36	19.2	17	42.8	8	9.9	4.9	10.2	112	13	1	1^D^
	18.3	16	42.8	8	9.9	4.9	10.2	110^e^	13	1	1^E^
	18.3	16	36.8	8	9.9	4.9	10.2	104.1	13	1	2^D^
	19.2	16.1^f^	41.8	8	9.9	4.9	10.2	110.1	13	1	2^E^
	30.8	17	34.7	8	10.8	4.9	10.2	117.4	17	1	8^E^
	31.8	17.1	34.7	8	10.8	4.9	10.2	118.5	19	1	7^D^
	31.8	17	34.8	8	10.8	4.9	10.2	118.5	24	1	3^D^
	31.8	17	34.7	8	10.8	4.9	10.2	118.4	24	1	5^E^
	31.8	18.1^g^	35.8	8	10.8	4.9	10.2	120.6	24	1	8^E^

Differences found in the results after one or more repetitions: ^a^24.5; ^b^30.8; ^c^17.3; ^d^26.9; ^e^10.3; ^f^16; ^g^18

^E^ Strain isolated after 3 days of feces enrichment

^D^ Strain isolated after direct culture of the feces

### Analysis of the residents’ faecal microbiota by barcoded pyrosequencing

**Fig. 1:**
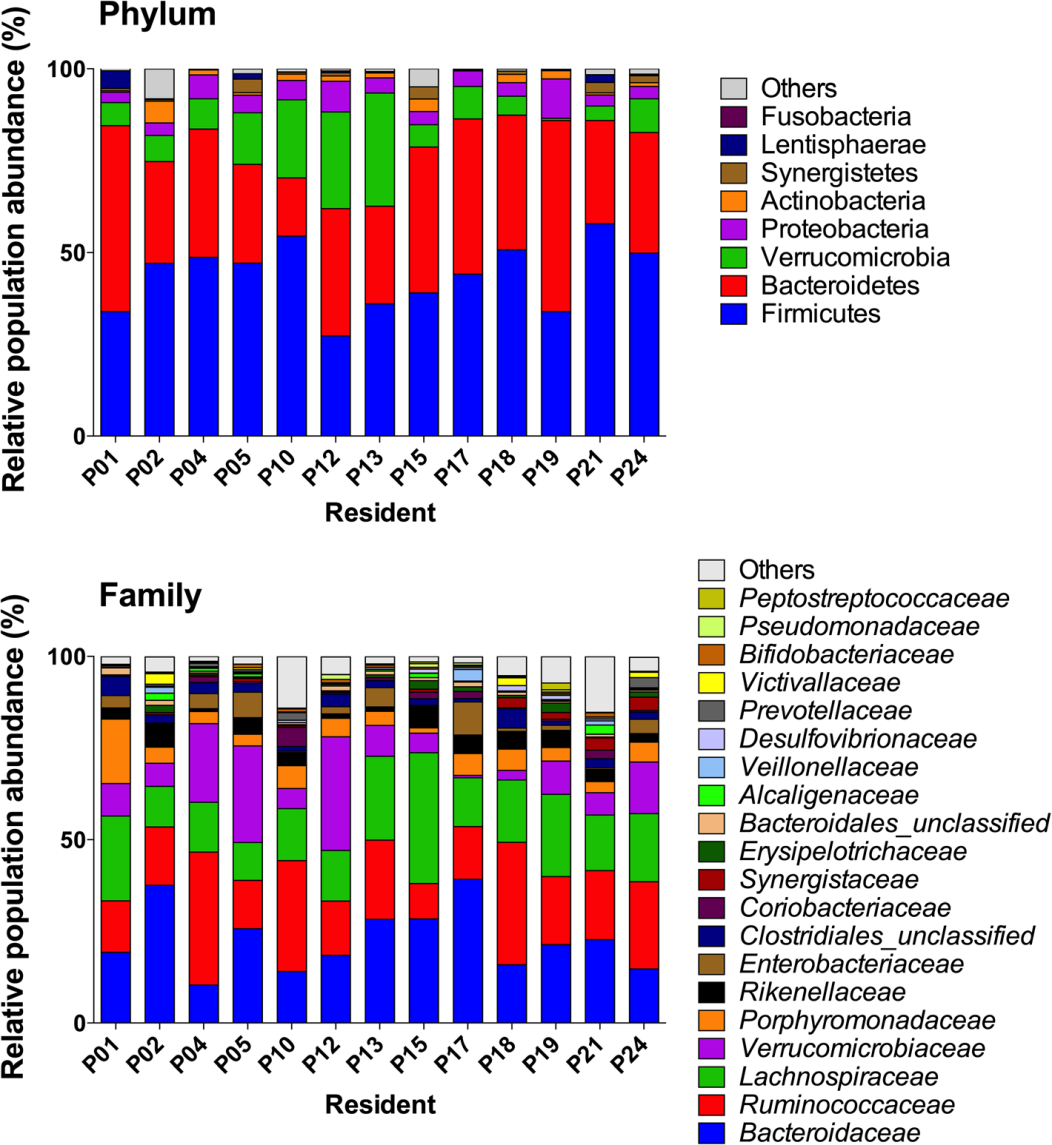
Taxonomical distribution deduced by 16S rRNA profiling. Bart chart detailing the mean cumulated relative abundance of the major phyla and families for each resident

Among the 23 residents, all available faecal samples from 13 residents (6 *C. difficile* negative and 7 *C. difficile* positive, in total 118 faecal samples) were selected for 16S profiling of their faecal microbiota. A total of 433,815 final reads were attributed to 3,940 species level OTUs (operational taxon units) among 118 samples (Additional file 1). The analysis showed that the major phyla found in patients were Firmicutes and Bacteroidetes followed by the Verrucomicrobia and the Proteobacteria (Fig. 1). On the family level, the major populations were consistent with previous human studies, *Bacteroidaceae*, *Ruminococcaceae* and *Lachnospiraceae* being dominant. The *Verrucomicrobiaceae, Porphyromonadaceae* and *Rikenellaceae* were subdominant (Fig. 1). The 6 major genera were *Bacteroides*, *Akkermansia*, *Parabacteroides*, *Alistipes* and two populations undefined at the genus level belonging respectively to the *Lachnospiraceae* and the *Ruminococcaceae* (Additional file 2).

**Fig. 2:**
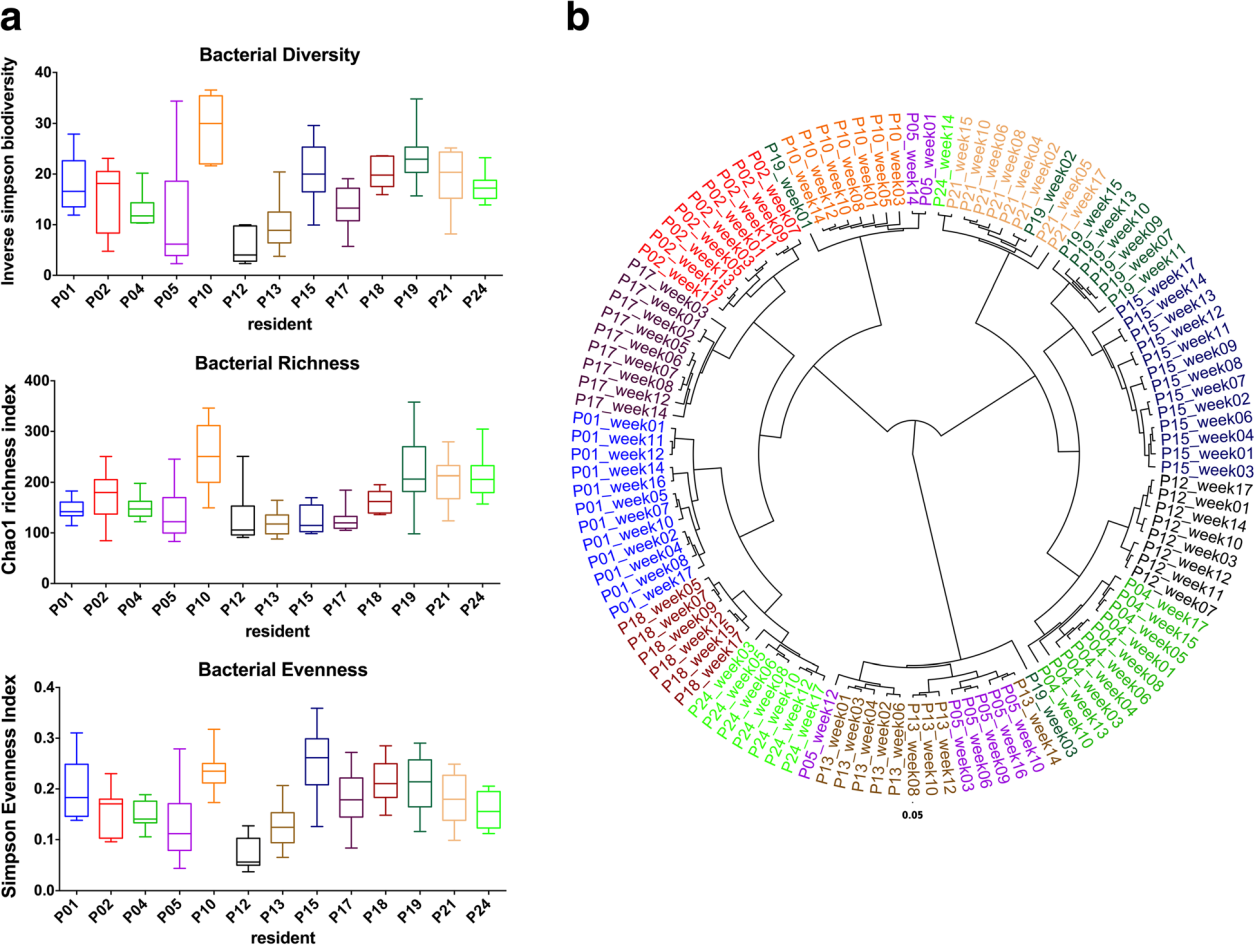
Species bacterial diversity and species phylotypic tree based on Bray-Curtis distance matrix. **a** Bacterial diversity (inverse Simpson biodiversity index), bacterial richness (Chao1 richness index) and bacterial evenness (Deduced from Simpson index). Bacterial diversity indexes are expressed as a box plot of the mean from subsampled datasets, whiskers represent minimum and maximum value. Median is shown as a line inside the box. **b** Phylotype tree of the 118 subsampled datasets built upon a Bray-Curtis distance matrix at the species taxonomical level (average tree is shown, 1000 iterations). The figure also shows all the faecal samples studied by 16S rDNA profiling analysis collected from each resident in different weeks

The mean alpha diversity and richness was variable between residents (Additional file 3), though no resident mean values are statistically different from the rest of the cohort (Fig. 2). Moreover, the analysis of the microbiota species structure and composition showed that each patient has his own microbiological imprint during the study as revealed by weighted UNIFRAC analysis of phylogenetic distribution of the samples based on a Bray-curtis distance matrix (Fig. 2 and Additional file 4).

**Fig. 3:**
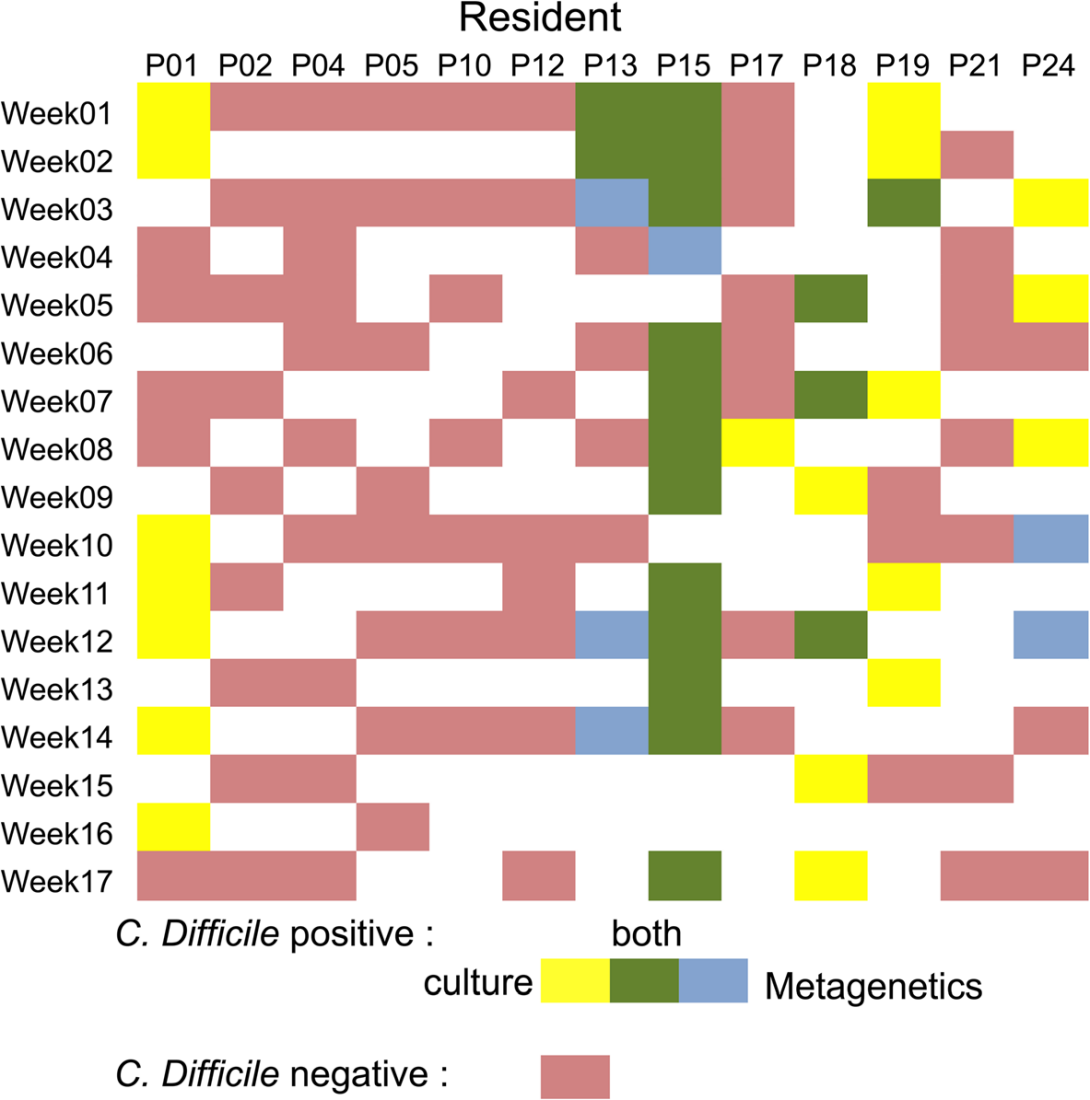
*C. difficile* detection results for the 118 samples analysed by culture and 16S rRNA gene analysis. Grid detailing the detection results for the samples analysed by both methods. For classic microbiology, samples are positive if either direct or enrichment culture is positive. For 16S profiling, samples are positive if at least one sequence read is identical to *C. difficile* V1-V3 16S rDNA sequence. Red – negative sample; white – non-analysed sample; yellow – *C. difficile* positive culture; blue – *C. difficile* sequence detection and green – *C. difficile* positive for both methods

Among the 118 samples, 24 samples were detected positive for *C. difficile* by 16S rRNA gene analysis (Fig. 3). Reads sharing minimum 99 % of identity to the *C. difficile* 16S rRNA sequence were identified as *C. difficile*. Nearest known species (*Clostridium glycolicum*, *Terrisporobacter mayombei* and *Romboutsia lituseburensis*) share less than 99 % of nucleotide identity on the V1-V3 hypervariable region with *C. difficile* 16S rRNA sequence. Thirty-seven out of 118 samples analysed by 16S rRNA gene analysis were positive for *C. difficile* positive by classical microbiology. Among the positive samples, 18 samples were detected by both methods, 19 samples were positive only by culture and 6 were positive only by 16S rRNA profiling.

### Link between *C. difficile* colonisation and faecal microbiota

**Fig. 4:**
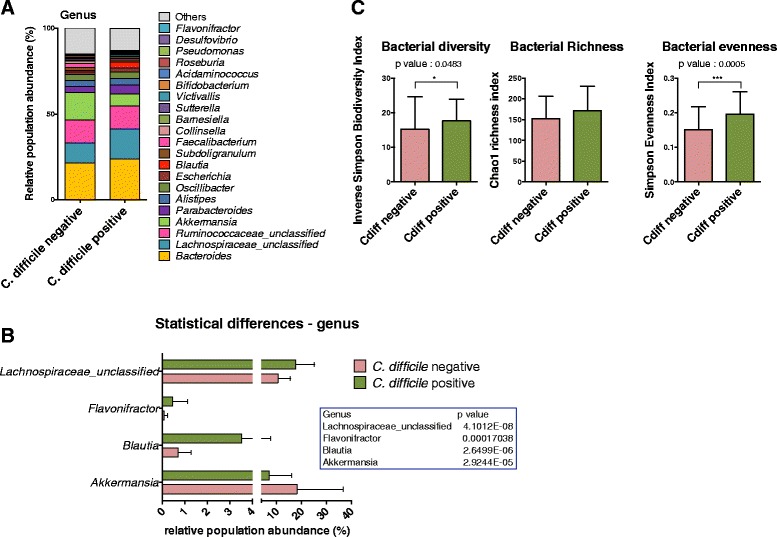
Microbiota comparison between *C. difficile* negative and positive residents. **a** Mean cumulative relative abundance distribution for the major bacterial genera (>1 %) for *C. difficile* negative (*N* = 38 samples) and positive residents (*N* = 80 samples). **b** Changes in microbial genus populations between *C. difficile* negative and positive residents. Populations whom relative abundance is statistically different are expressed as mean relative abundance ± standard error of the mean (*p* < 0.05 according to multiple unpaired t-test with Benjamini-Hochberg False Discovery Rate). **c** Bacterial diversity (inverse Simpson biodiversity index), bacterial richness (Chao1 richness index) and bacterial evenness (Deduced from Simpson index). Bacterial diversity indexes are expressed as the mean value with standard deviation, from subsampled datasets. Statistical differences are represented by asterisks (*p* < 0.05 according to non parametric Mann–Whitney test) after Bonferroni corrections

In order to explore the link between *C. difficile* colonisation and the resident microbiota, residents negative and positive for *C. difficile* were grouped. As the inter-individual variability is the main driving factor for the sample clustering, the grouping was made by resident instead of strict positive and negative samples. Figure 4 shows the major mean genus relative abundance for both groups. Statistical analysis revealed that only four genus populations have significant relative abundance between both groups (Fig. 4). *Blautia* (Firmicutes) and *Flavonifractor* (Firmicutes) and the Lachnospiraceae_unclassified (Firmicutes) appeared more abundant in the *C. difficile* positive group, whereas *Akkermansia* (*Verrucomicrobiaceae*) abundance was higher in the *C. difficile* negative group. In order to better understand these differences, both groups were further divided into diarrhoeic (>1 diarrhoeic faeces sample) or non-diarrhoeic residents. Results showed a decrease in *Verrucomicrobiaceae* linked to *C. difficile* positive groups (data not shown). In addition, a higher abundance of *Lachnospiraceae* family was detected in *C. difficile* positive diarrhoeic residents compared to other groups (*p* < 0.05) (Additional file 5).

The analysis of mean alpha diversity of both groups showed that *C. difficile* positive group does not exhibited an increased faecal microbial diversity compared to *C. difficile* negative group after Bonferroni corrections (Fig. 4). No differences were revealed in the species richness (Chao index). The diarrhoeic status does not appear to have an influence on the results obtained (data not shown).

## Discussion

The gut microbiota ecosystem plays a critical role in resistance to colonisation by pathogenic organisms, infection and recurrence [11]. *C. difficile* colonisation has been described as ten times higher in elderly nursing home residents than in the general population living outside long-term care facilities [13]. The deteriorating health status of nursing home residents, their frequent hospitalisation and the cohabitation in the same contaminated environment promote bacterial colonisation and dissemination [14, 15]. The aim of this study was to evaluate the presence of *C. difficile* in a short cohort of elderly nursing home residents and to evaluate the global evolutions of their faecal microbiota.

In the present study, 30.4 % (7/23) were positive to *C. difficile*. In previous studies conducted in Germany, United Kingdom, Ireland, Australia or Canada, the prevalence of positive residents reported ranges between 0.80 and 10 % [13, 16–19]. This prevalence is much higher in other reports in USA, varying between 6.4 and 54.8 %. The same scenario was reported for the incidence of CDI in Belgian hospitals when compared with other hospitals in Europe and USA [20, 21]. In this study, only one resident was diagnosed with a CDI. However, other residents presented symptoms (diarrhoea) and either stool test positive for toxigenic *C. difficile*. Therefore, the lack of clinical diagnosis or request do not exclude that other residents suffered CDI during the study period. On the other hand, positive residents to *C. difficile* without any signs of disease were also detected. Results obtained from PCR-ribotyping and MLVA showed that there was a clonal dissemination within the nursing home residents. Therefore, even if some authors have refuted the theory of person-to person transmission to explain the increase incidence of CDI within hospital awards [22, 23], it seems that in nursing homes the situation is different. Only four different PCR-ribotypes were identified and three of them were toxigenic (UCL16a, UCL46 and 027). Surprisingly, none of them were among the five PCR-ribotypes most commonly identified in Belgian hospitals in 2013 and 2014 [21]. Since 2011, decline in the prevalence of the PCR-ribotype 027 has been reported in different European countries. Furthermore, in Belgium, the proportion of hospitals with the hypervirulent PCR-ribotype 027 decreases from 34 % in 2009 to 15 % in 2013 [20]. Nursing home population is closed and restricted and changes in the prevalence of PCR-ribotypes come later than in hospitals. Therefore, it could be hypothesised that the most prevalent PCR-ribotypes today in hospitals (PCR-ribotypes 078 and 014/020) [21] will be in a few years predominant in nursing homes. In previous studies on elderly gut microbiota, Bacteroidetes and Firmicutes have been reported to dominate, with a marked preponderance of Bacteroidetes over Firmicutes [24–26]. In the present study, the major bacterial phyla identified in residents’ microbiota are Firmicutes followed by Bacteroidetes. We also found a higher abundance of Verrucomicrobia than previously observed [27, 28]. The predominance of Firmicutes and Bacteroidetes has also been highlighted in a large cohort study in Belgium [27], although the overall prevalence of Bacteroidetes in our study is higher than the mean value on a large-scale population level (34 % in our study vs 25 % in the Belgian Flemish Gut Flora Project, unpublished data). This increase in Bacteroidetes relative proportion in elderly gut microbiota compared to a matched cohort of younger adults has already been described [27].

It has been recently underlined that longitudinal survey of microbiota in elderly and long-stay residents did not support a model of unstable microbiota and diversity [28]. The longitudinal analysis of the bacterial diversity of community composition showed that bacterial diversity and richness is variable between residents but did not reveal any evolution during the study. Moreover, inter-individual microbiota variability is known to be greater than temporal variability [25] and has been confirmed by community structure analysis.

There are a growing number of publications on the gut microbiota exploration and CDI. Some of them focus on the idea that commensal bacterial populations can protect from CDI [29]. Although no candidate population has emerged, loss of some bacterial genera like *Bacteroides* has been associated with CDI [30]. Other studies on hospitalized CDI patients described a significant alteration of gut microbiota during CDI along with decreased biodiversity and richness [29, 30]. This alteration includes a rise in *Proteobacteria* and a decrease in *Lachnospiraceae* and other butyrate-producing bacteria [29]. However, it should be noted that these alterations do not appear to be specific to CDI and are also observed in patients without *C. difficile* diarrhoea. In a first extensive study on elderly and CDI, Rea et al. [30] showed that there was little difference regarding the microbiota composition between CDI subjects and asymptomatic *C. difficile* carriers. Moreover, only minor bacterial taxon showed a statistically different abundance between *C. difficile* positive subjects and negative individuals.

The 16S rRNA profiling has been performed on a limited cohort of *C. difficile* negative and positive residents. Even if it was longitudinal, we did not focus on the pathology or on the antibiotic use that might have occurred during the survey. We centred this analysis on the hypothesis that in these long term stay residents, *C. difficile* persistent or recurrent colonisation might be associated with more pronounced differences in microbiota between both groups. Significant changes have been observed in *C. difficile* positive individuals in the relative abundance of bacterial populations, but these are limited to the *Lachnospiraceae* and *Verrucomicrobiaceae*. Surprisingly, *Lachnospiraceae* and specifically genus *Blautia* abundance is higher in *C. difficile* positive individuals, which is quite different from previous reports [31, 32]. Even if CDI diagnosis was not specifically performed during the study, we further split both residents groups regarding the presence of diarrhoeic faeces and observed that this bacterial family abundance is significantly higher in *C. difficile* positive residents having diarrhoeic faeces compared to diarrhoeic *C. difficile* negative individuals. *Verrucomicrobiaceae* (genus *Akkermansia*) is known to be linked to gut health and its abundance seems to be reduced in context of gut inflammation [33]. In addition, *Akkermansia* is an appealing candidate to become a human probiotic, selected based on established mechanisms of preventive treatment of obesity and diabetes [34, 35]. Even if gut inflammatory status of the residents has not been investigated, it is a known risk factor for *C. difficile* colonisation and could therefore be responsible for this negative correlation.

Positive *C. difficile* status is not associated with microbiota richness or biodiversity reduction in our study (failed significance after Bonferroni corrections). It appears that impact on gut microbiota structure is associated with actual diarrhoeic episodes instead of *C. difficile* positive status [30]. Recent studies have demonstrated that stool consistency is a dominant factor associated with microbiota composition and negatively correlate species richness with stool looseness [27, 34]. Comparison between diarrhoeic samples versus non-diarrhoeic samples in this study confirms this with a slight yet significant decrease in bacterial richness (*p* < 0.05 - data not shown) but not in biodiversity.

This study underlines that *C. difficile* status in long-term stay elderly resident is associated with specific changes in microbiota composition. We confirm that the detection alone of this bacterium cannot be linked to major changes in microbiota structure. We have shown that longitudinal study underlines the dynamic of *C. difficile* status and the relative stability of gut microbiota in these elderly populations. The major limitation of this is the relatively low number of volunteers. Microbiota analysis has been marked by a strong inter-individual variability, which can certainly influence comparisons between *C. difficile* negative and positive groups. It has been recently shown that several types of microbiota composition might increase susceptibility to CDI [31]. Further studies on long-term stay residents will be needed to improve our knowledge of the *C. difficile* reservoir and susceptibility in nursing homes.

## Conclusions

*C. difficile* colonisation is higher in nursing homes than in hospitals, with a predominance of the hypervirulent PCR-ribotype 027. MLVA reveals a clonal dissemination of this PCR-ribotype among nursing home residents. In the last years the prevalence of this type is decreasing in hospitals, suggesting that the isolation of elderly in nursing homes has an important impact on the type of strains found. Changes were observed in *C. difficile* positive individuals in the relative abundance of some bacterial populations, including *Lachnospiraceae* and *Verrucomicrobiaceae. Lachnospiraceae* and specifically genus *Blautia* abundance is higher in *C. difficile* positive individuals than in negative individuals. Positive *C. difficile* status is not associated with microbiota richness or biodiversity reduction in this study. Notably, a decrease of *Akkermansia* in positive subjects was repeatedly found. The link between *Akkermansia,* gut inflammation and *C. difficile* colonisation merits further investigations.

## Methods

### Resident recruitment and sampling

The study was conducted at the Saint-Joséphine (ACIS) nursing home, in the province of Liège (Theux), Belgium. This local nursing home has a total capacity of 110 beds with a total of 73 employees. Data collected included gender, age, clinical status, medical history, recent history of diarrhoea, recent hospitalization, medication, including non-steroidal anti-inflammatory drugs (NSAIDs) or antibiotics, probiotics and changes in diet.

During a 4-month period, from March through June 2013, stool samples from a group of 23 elderly care home residents were collected weekly. Most of the subjects were aged 65 years and older. Faecal sampling was performed from Thursday until early Friday. Two samples per person were collected. The first sample was collected in an individual identified sterile 50 ml tube for further culture to detect *C. difficile.* The second one was collected using the Stool DNA Stabilizer (PSP^R^ Spin Stool DNA Plus Kit 00310; Invitek, Westburg b.v., Netherlands) to study the microbial biodiversity of the faeces content by amplicon sequencing. Samples obtained were scored as normal, diarrhoea or bloody diarrhoea faeces. They were kept at 4 °C for a maximum of 48 h until their arrival in the laboratory for immediate culture or DNA extraction.

### *C. difficile* culture, identification and characterisation

Culturing of faeces (with and without a phase of enrichment), isolation and identification of *C. difficile* colonies were performed as previously described [36]. Toxin activity of the isolated strains was confirmed by a cytotoxicity assay using confluent monolayer MRC-5 cells as described previously [37].

### Molecular typing of *C. difficile* isolates

*C. difficile* isolates were tested using Genotype Cdiff system (Hain Lifescience, Nehren, De) for the presence of the *tpi* gen, toxin genes *tcdA*, *tcdB*, *cdtA* and *cdtB*, deletions in the regulator gene *tcdC* and *gyrA* mutation, according to the manufacturer’s instructions.

PCR-ribotyping was performed using the primers and conditions described by Bidet et al. [38]. An international number was used for *C. difficile* strains that presented a PCR-ribotype profile matching the Cardiff ribotypes from the strain collection available in our laboratory. Otherwise, strains were identified with an internal nomenclature.

### MLVA

The DNA extraction was performed using a chelex 100 solution 5 % (Biorad, Nazareth, Be) as described previously [39]. For MLVA, seven VNTR loci (A6, B7, C6, E7, G8, CDR5, CDR60) were studied as previously described [40]. Isolates with MLVA STRD ≤ 2 were indicative of a high degree of genetic relatedness [41].

### 16S rRNA pyrosequencing and data analysis

Total bacterial DNA was extracted from the stool samples with the PSP® Spin Stool DNA Plus Kit 00310 (Invitek), following the manufacturer’s recommendations. 16S rRNA profiling, targeting V1-V3 hypervariable region and sequenced on Roche GS Junior was performed as described previously [36]. Briefly, libraries from 20 samples were run in the same titanium pyrosequencing reaction using Roche multiplex identifiers, and amplicons were sequenced using the Roche GS-Junior Genome Sequencer instrument (Roche). A total of six sequencing runs were necessary to obtain the data for the 118 samples.

Sequence reads processing was treated as previously described [37] using respectively MOTHUR software package v1.35, Pyronoise algorithm and UCHIME algorithm for alignment and clustering, denoising and chimera detection (MOTHUR script has been added as Additional file 6) [42–44]. 16S rRNA Reference alignment and taxonomical assignation in MOTHUR were based upon the SILVA database (v1.15) of full-length 16S rRNA sequences [45]. Clustering distance of 0.03 was used for OTU generation. Subsample datasets were obtained and used to evaluate ecological indicators, Richness estimation (Chao1 estimator), microbial biodiversity (reciprocal Simpson index), and the population evenness (derived from Simpson index) at the phylotype species level using MOTHUR. Population structure and community membership were assessed with MOTHUR using distance matrice based on Bray-Curtis dissimilarity index (a measure of community structure which considers shared OTUs and their relative abundances [46, 47] abundances).

Weighted UNIFRAC test implemented in MOTHUR v1.35 was used to assess differences regarding bacterial community structure between residents. Statistical differences in bacterial biodiversity, richness and evenness between residents and between *C. difficile* positive and *C. difficile* negative groups were respectively assessed using one way-ANOVA and Mann–Whitney test using PRISM 6 (Graphpad Software). In order to highlight statistical differences in the bacterial population abundance between groups, multiple unpaired *t*-test with Benjamini-Hochberg False Discovery Rate were performed using PRISM 6 (Graphpad Software). Differences were considered significant for a *p*-value of less than 0.05, adjusted with Bonferroni corrections.

## Additional files

Additional file 1: Quality analysis of the 16S rRNA gene analysis for the 118 human faecal samples. (DOCX 40 kb) Additional file 2: Taxonomical distribution deduced by 16S rRNA profiling. Bart chart detailing the mean cumulated relative abundance of the major genera for each resident. Bart chart detailing the mean cumulated relative abundance of the major genera for each resident. (PNG 3354 kb) Additional file 3: Longitudinal distribution of the ecological indicators. Bacterial diversity (inverse Simpson biodiversity index), bacterial richness (Chao1 richness index) and bacterial evenness (Deduced from Simpson index) expressed for each analysed samples. (PNG 4002 kb) Additional file 4: Unifrac weighted score and significance between patients. UNIFRAC weighted score (W score) and significance for patients clustering based on Bray-Curtis dissimilarity distance matrix. (DOCX 84 kb) Additional file 5: Relative abundance of *Lachnospiraceae* between groups of diarrhoeic/non diarrhoeic and *C. difficile* status. *Lachnospiraceae* relative abundance is expressed as mean relative abundance ± standard error of the mean. Different superscript letters correspond to statistical difference according to one way ANOVA with Tukey-Kramer post-hoc test (*p* < 0.05). (PNG 1052 kb) Additional file 6: MOTHUR script used for 16S rRNA gene analysis. (TXT 1 kb)

## Abbreviations

*C. difficile*: *Clostridium difficile*
CDI: *C. difficile* infection
MLVA: Multilocus variable number of tandem repeats analysis
NSAIDS: Non-steroidal anti-inflammatory drugs
OTUs: Operational taxon units
STRD: Summed tandem repeat differences
VNTR: Variable number tandem repeat
