# Supplementary material for: Longitudinal survey of Clostridium difficile presence and gut microbiota composition in a Belgian nursing home
Source: BMC Microbiol. 2016 Oct 1;16:229. doi: 10.1186/s12866-016-0848-7 (PMC5045619; doi:10.1186/s12866-016-0848-7)
Supplement: Additional file 1: — Quality analysis of the 16S rRNA gene analysis for the 118 human faecal samples. (DOCX 40 kb) [file 12866_2016_848_MOESM1_ESM.docx]

Additional file 1. Quality analysis of the metagenetic libraries created for the 118 human faecal samples analysed.

| Item | Total number (or %) | Mean read length, nucleotide |
| --- | --- | --- |
| Raw reads | 590,067 | 512 |
| Postdenoising/Postchimeric | 433,815 | 454 |
| Loss in Treatment (%) | 26 |  |
| OTU 0.03^a^ | 10,458 |  |
| Phylotype species | 3940 |  |
| Phylotype genus | 208 |  |

^a^ OTU clustering distance
