# Supplementary figures and images for: Longitudinal survey of Clostridium difficile presence and gut microbiota composition in a Belgian nursing home
Source: BMC Microbiol. 2016 Oct 1;16:229. doi: 10.1186/s12866-016-0848-7 (PMC5045619; doi:10.1186/s12866-016-0848-7)

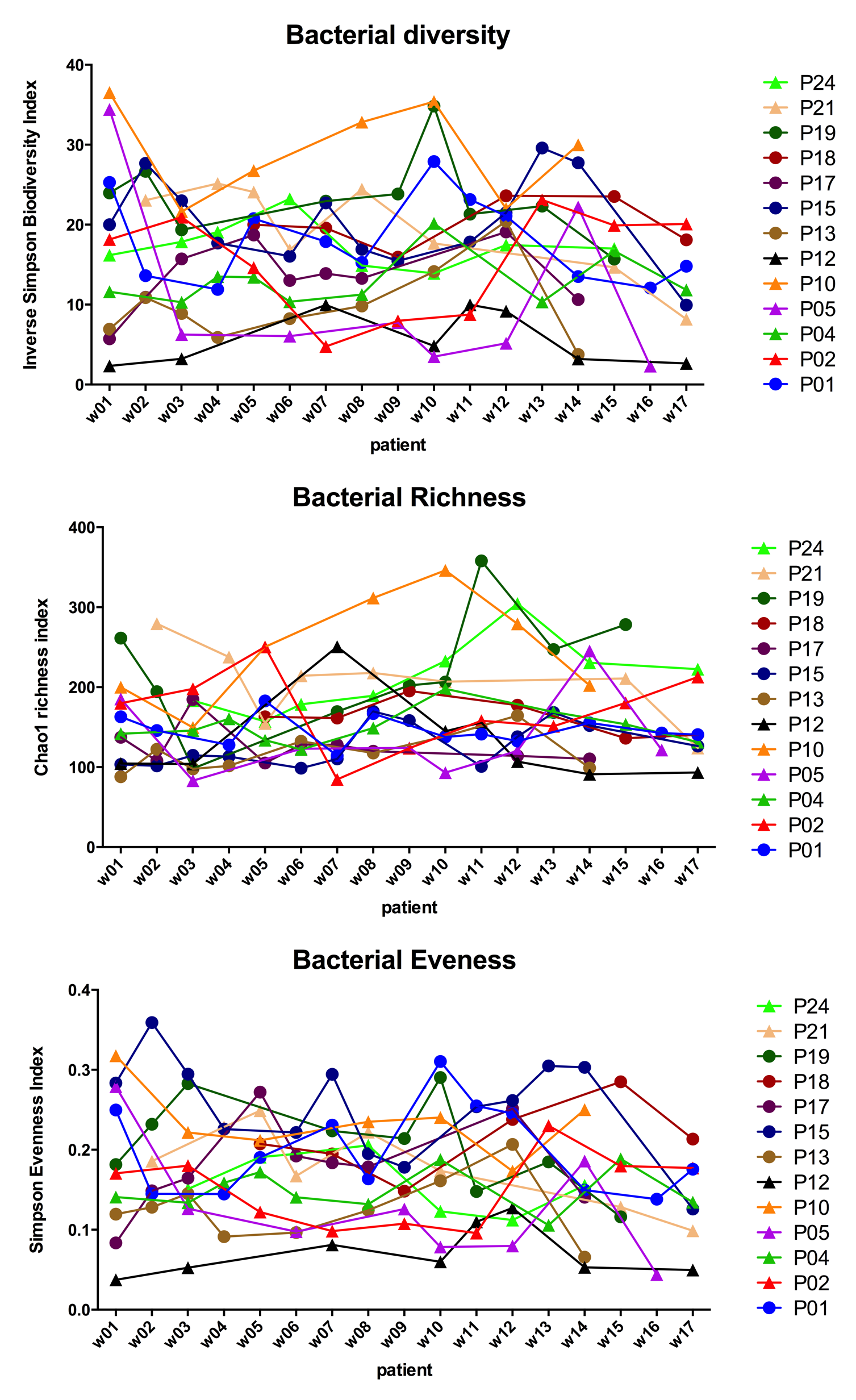

Supplement: Additional file 3: — Longitudinal distribution of the ecological indicators. Bacterial diversity (inverse Simpson biodiversity index), bacterial richness (Chao1 richness index) and bacterial evenness (Deduced from Simpson index) expressed for each analysed samples. (PNG 4002 kb) [file 12866_2016_848_MOESM4_ESM.png]

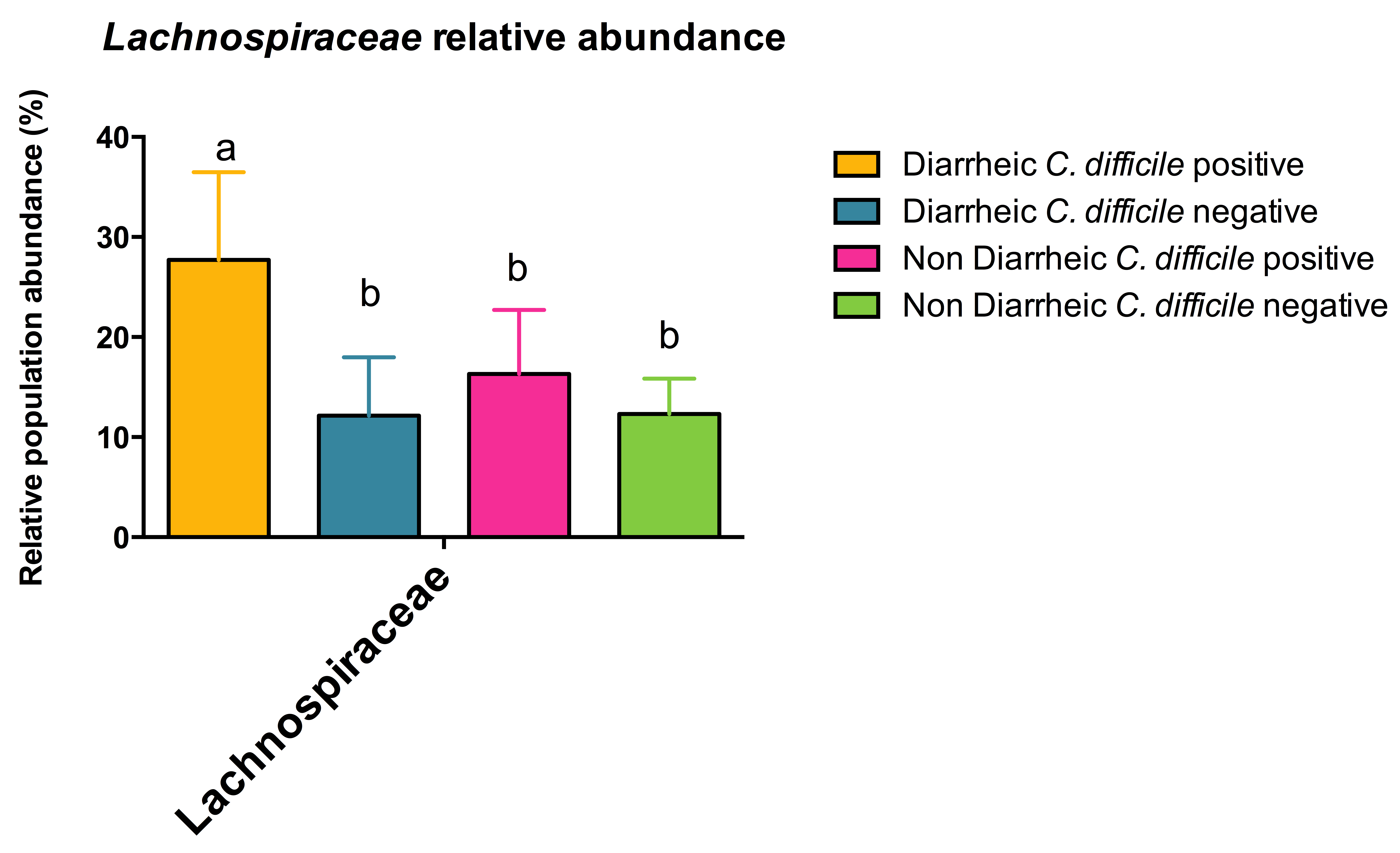

Supplement: Additional file 5: — Relative abundance of Lachnospiraceae between groups of diarrhoeic/non diarrhoeic and C. difficile status. Lachnospiraceae relative abundance is expressed as mean relative abundance ± standard error of the mean. Different superscript letters correspond to statistical difference according to one way ANOVA with Tukey-Kramer post-hoc test (p < 0.05). (PNG 1052 kb) [file 12866_2016_848_MOESM5_ESM.png]
