# Supplementary material for: Longitudinal survey of Clostridium difficile presence and gut microbiota composition in a Belgian nursing home
Source: BMC Microbiol. 2016 Oct 1;16:229. doi: 10.1186/s12866-016-0848-7 (PMC5045619; doi:10.1186/s12866-016-0848-7)
Supplement: Additional file 4: — Unifrac weighted score and significance between patients. UNIFRAC weighted score (W score) and significance for patients clustering based on Bray-Curtis dissimilarity distance matrix. (DOCX 84 kb) [file 12866_2016_848_MOESM3_ESM.docx]

| W Score | |  |  |  |  |  |  |  |  |  |  |  |
| --- | --- | --- | --- | --- | --- | --- | --- | --- | --- | --- | --- | --- |
|  | P01 | P02 | P04 | P05 | P10 | P12 | P13 | P15 | P17 | P18 | P19 | P21 |
| P01 |  |  |  |  |  |  |  |  |  |  |  |  |
| P02 | 1 |  |  |  |  |  |  |  |  |  |  |  |
| P04 | 1 | 1 |  |  |  |  |  |  |  |  |  |  |
| P05 | 0.989227 | 0.989227 | 0.994597 |  |  |  |  |  |  |  |  |  |
| P10 | 1 | 1 | 1 | 0.975752 |  |  |  |  |  |  |  |  |
| P12 | 1 | 1 | 1 | 0.994597 | 1 |  |  |  |  |  |  |  |
| P13 | 1 | 1 | 1 | 0.811885 | 1 | 1 |  |  |  |  |  |  |
| P15 | 1 | 1 | 1 | 0.994597 | 1 | 1 | 1 |  |  |  |  |  |
| P17 | 1 | 1 | 1 | 0.989227 | 1 | 1 | 1 | 1 |  |  |  |  |
| P18 | 1 | 1 | 1 | 0.989227 | 1 | 1 | 1 | 1 | 1 |  |  |  |
| P19 | 0.978402 | 0.978402 | 0.91147 | 0.987315 | 0.976086 | 0.91147 | 0.997421 | 0.898462 | 0.978402 | 0.978402 |  |  |
| P21 | 1 | 1 | 1 | 0.994597 | 1 | 1 | 1 | 1 | 1 | 1 | 0.893297 |  |
| P24 | 0.953487 | 0.969149 | 0.996232 | 0.989227 | 0.980789 | 0.996232 | 1 | 0.996232 | 0.969149 | 0.905936 | 0.937105 | 0.93807 |

| W significance | |  |  |  |  |  |  |  |  |  |  |  |
| --- | --- | --- | --- | --- | --- | --- | --- | --- | --- | --- | --- | --- |
|  | P01 | P02 | P04 | P05 | P10 | P12 | P13 | P15 | P17 | P18 | P19 | P21 |
| P01 |  |  |  |  |  |  |  |  |  |  |  |  |
| P02 | <0.001 |  |  |  |  |  |  |  |  |  |  |  |
| P04 | <0.001 | <0.001 |  |  |  |  |  |  |  |  |  |  |
| P05 | <0.001 | <0.001 | <0.001 |  |  |  |  |  |  |  |  |  |
| P10 | <0.001 | <0.001 | <0.001 | <0.001 |  |  |  |  |  |  |  |  |
| P12 | <0.001 | <0.001 | 0.011 | <0.001 | <0.001 |  |  |  |  |  |  |  |
| P13 | <0.001 | <0.001 | 0.076 | 0.075 | <0.001 | 0.040 |  |  |  |  |  |  |
| P15 | <0.001 | <0.001 | <0.001 | <0.001 | <0.001 | <0.001 | <0.001 |  |  |  |  |  |
| P17 | <0.001 | <0.001 | <0.001 | <0.001 | <0.001 | <0.001 | <0.001 | <0.001 |  |  |  |  |
| P18 | <0.001 | <0.001 | <0.001 | <0.001 | <0.001 | <0.001 | <0.001 | <0.001 | <0.001 |  |  |  |
| P19 | <0.001 | <0.001 | <0.001 | <0.001 | <0.001 | <0.001 | <0.001 | <0.001 | <0.001 | <0.001 |  |  |
| P21 | <0.001 | <0.001 | <0.001 | <0.001 | <0.001 | <0.001 | <0.001 | <0.001 | <0.001 | <0.001 | <0.001 |  |
| P24 | <0.001 | <0.001 | <0.001 | <0.001 | <0.001 | <0.001 | <0.001 | <0.001 | <0.001 | <0.001 | <0.001 | <0.001 |
